# Managing uncertainty - a qualitative study of surgeons’ decision-making for one-stage and two-stage revision surgery for prosthetic hip joint infection

**DOI:** 10.1186/s12891-017-1499-z

**Published:** 2017-04-12

**Authors:** Andrew J. Moore, Ashley W. Blom, Michael R. Whitehouse, Rachael Gooberman-Hill

**Affiliations:** School of Clinical Sciences, University of Bristol, Bristol, UK

**Keywords:** Decision-making, Prosthetic joint infection, Hip arthroplasty, Hip replacement, Orthopaedic surgery, Qualitative

## Abstract

**Background:**

Approximately 88,000 primary hip replacements are performed in England and Wales each year. Around 1% go on to develop deep prosthetic joint infection. Between one-stage and two-stage revision arthroplasty best treatment options remain unclear. Our aims were to characterise consultant orthopaedic surgeons’ decisions about performing either one-stage or two-stage revision surgery for patients with deep prosthetic infection (PJI) after hip arthroplasty, and to identify whether a randomised trial comparing one-stage with two-stage revision would be feasible.

**Methods:**

Semi-structured interviews were conducted with 12 consultant surgeons who perform revision surgery for PJI after hip arthroplasty at 5 high-volume National Health Service (NHS) orthopaedic departments in England and Wales. Surgeons were interviewed before the development of a multicentre randomised controlled trial. Data were analysed using a thematic approach.

**Results:**

There is no single standardised surgical intervention for the treatment of PJI. Surgeons balance multiple factors when choosing a surgical strategy which include multiple patient-related factors, their own knowledge and expertise, available infrastructure and the infecting organism. Surgeons questioned whether it was appropriate that the two-stage revision remained the best treatment, and some surgeons' willingness to consider more one-stage revisions had increased over recent years and were influenced by growing evidence showing equivalence between surgical techniques, and local observations of successful one-stage revisions. Custom-made articulating spacers was a practice that enabled uncertainty to be managed in the absence of definitive evidence about the superiority of one surgical technique over the other. Surgeons highlighted the need for research evidence to inform practice and thought that a randomised trial to compare treatments was needed. Most surgeons thought that patients who they treated would be eligible for trial participation in instances where there was uncertainty about the best treatment option.

**Conclusions:**

Surgeons highlighted the need for evidence to support their choice of revision. Some surgeons' willingness to consider one-stage revision for infection had increased over time, largely influenced by evidence of successful one-stage revisions. Custom-made articulating spacers also enabled surgeons to manage uncertainty about the superiority of surgical techniques. Surgeons thought that a prospective randomised controlled trial comparing one-stage with two-stage joint replacement is needed and that randomisation would be feasible.

**Electronic supplementary material:**

The online version of this article (doi:10.1186/s12891-017-1499-z) contains supplementary material, which is available to authorized users.

## Background

Approximately 88,000 primary hip replacements were performed in England and Wales during 2014 [1]. After surgery, about 1% of patients subsequently develop deep prosthetic joint infection (PJI) [2]. Treatment options for early PJI can include non-surgical antimicrobial therapy, or débridement with prosthesis retention. For more established infections a prosthesis exchange is required of which there are two types [3]. In a one-stage process, the prosthesis is removed, the area debrided and a new prosthesis is inserted immediately [4, 5]. In a two-stage process, after removal of implants and debridement there is often an interval period of around 3–6 months during which systemic antibiotic therapy is delivered and can be augmented by local delivery from a spacer containing antibiotic loaded cement. A definitive prosthesis is then implanted during a second operation [5–7]. A variation on these one-stage or two-stage strategies is the use of a custom-made articulating spacer (CUMARS). This approach aims to provide the patient with a hip joint between stages that can bear weight and provides reasonable function. This technique involves the removal of the implant and insertion of a new implant that is “loosely” cemented into place. Although this first operation is intended to be the first of a two-stage process, if the implant provides the patient with sufficient function then a further operation is not conducted. Examples of this method include the PROSTALAC [8] and CUMARS hip [9]. Cases of CUMARS hip replacements remaining in situ at 12 years follow-up have been reported [9].

All revision for infection has great impact on patients [10], but patients undergoing two-stage revision report that the period in between operations is particularly difficult, with reduced mobility and poor quality of life at this time [10, 11]. Although two-stage revision provides the opportunity for additional antimicrobial strategies [12], around half of patients experience complications associated with spacers [13] and the cost of a two-stage revision for hip PJI is 1.7 times that of a one-stage revision [14, 15]. Currently there is no adequately powered randomised trial to determine the optimum treatment strategy in PJI of the hip. The most recent high quality systematic reviews in unselected patients indicate that re-infection rates are relatively similar in one-stage and two-stage revision [16, 17]. There is a paucity of evidence regarding patient quality of life and satisfaction for the different treatment strategies for PJI of the hip [18, 19]. Despite the fact that two-stage revision is associated with higher surgical morbidity and mortality rates [20], it remains more commonly performed than one-stage revision for PJI of the hip (one-stage: 30%; two-stage: 64%; excision: 6% [21].

The design of high quality randomised controlled trials (RCT) benefits from careful planning to consider factors that might impede or facilitate recruitment and adherence to protocols [22]. In any trial to compare surgical treatments for PJI, there is a need to consider not only how patients might view a trial, but also whether surgeons are willing to be involved in a trial that necessarily involves random allocation of patients to either one-stage or two-stage surgery. This is particularly the case for PJI because of the complex decisions that surgeons make when deciding on what treatment is most appropriate. A first step in trial design is identifying how these complex decisions are made in current practice. This information enables trial designers to establish feasibility of a randomised trial and to inform elements of trial design.

To date no research has characterised how surgeons make decisions about providing one-stage or two-stage revision treatment for PJI. Understanding decision-making requires attention to the detail and rationale underpinning those decisions. A robust way to achieve this is the use of qualitative research methods [23], which are increasingly used to inform trial design [24, 25], are recommended by the Medical Research Council [22] and have demonstrable value across specialties including orthopaedic surgery [26]. Using qualitative methods, we characterised how orthopaedic surgeons decided to use either one-stage or two-stage revision arthroplasty for hip PJI and to assess the feasibility and inform the design of a future randomised trial.

## Methods

### Setting and sampling

This qualitative interview study employed purposive sampling and the sample size was designed to achieve data saturation. Purposive sampling is used to ensure that participants have experience of relevance to the study question [26, 27] and data saturation is achieved whereby collecting more data would not achieve further insight [28]. An appropriate sample for this study comprised consultant orthopaedic surgeons who were making decisions about treatment for hip PJI, and who regularly conducted both one-stage and two-stage revision. The sample size of 12 surgeons, as described below, was adequate because data saturation was achieved. Twenty-three surgeons from 5 high-volume National Health Service (NHS) orthopaedic departments treating PJI were invited to participate. A member of the study team contacted each centre to identify eligible surgeons and sent them an information pack, which included a letter of invitation, information booklet, reply form and pre-paid envelope. Surgeons who returned a reply form that expressed their interest in taking part in the study were contacted by a researcher who then made arrangements for a face-to-face or telephone interview. All surgeons provided their written informed consent before interview and all agreed to be audio-recorded and to the publication of anonymised quotations from the interviews. Ethics approval for the study was granted by NRES (National Research Ethics Service) Committee South West —Exeter (14/SW/0072) on 29 April 2014.

### Data collection and analysis

We conducted interviews with 12 orthopaedic surgeons, either by telephone (*n* = 10) or face-to-face in an available room at hospital (*n* = 2). The average age of the participants was 49 years and all were men. Average number of years treating infection was 14 (ranging from 3 to 23), see Table 1. Interviews were conducted using a semi-structured topic guide, focused on treatment of PJI, important outcomes, and views about a future RCT [see Additional file 1]. The interviews were conducted by an experienced qualitative researcher (AJM), and lasted for an average of 29 min (ranging from 16 to 44 min). Interviews were audio-recorded, transcribed and anonymised.

**Table 1:** Participant characteristics

No.	Pseudonym	Treatment centre	Age (range in years)	Years treating PJI (range in years)	Revision type self-reported as performed most often (one-stage, two-stage or CUMARS)
1	Steven	Centre 1	35–50	0–10	Two-stage
2	Dave	Centre 1	35–50	0–10	Two-stage
3	Tom	Centre 2	35–50	0–10	CUMARS
4	Howard	Centre 2	35–50	20+	CUMARS
5	Harrison	Centre 3	35–50	0–10	CUMARS
6	James	Centre 3	51–65	20+	Equal for one- and two-stage
7	Tim	Centre 3	51–65	10–19	Two-stage
8	Carl	Centre 3	51–65	10–19	CUMARS
9	Brian	Centre 4	51–65	10–19	CUMARS
10	Duncan	Centre 4	51–65	20+	CUMARS
11	Alex	Centre 5	51–65	10–19	Two-stage
12	Saul	Centre 5	35–50	10–19	Two-stage
			Average 49	Average 14	

The data were analysed using a thematic method [29], which involved reading and re-reading the transcripts, followed by initial inductive coding and grouping of the data into themes and subthemes. Themes were further refined to ensure internal and external coherence (fit within the theme and externally within the whole data set) [29]. To ensure rigour, four transcripts were independently double-coded by another experienced qualitative researcher (RG-H), and codes discussed, agreed and then applied to the data set with ongoing refinement as needed. Data collection and analysis took place concurrently and data collection stopped once thematic saturation was achieved [28]. All names are pseudonyms.

## Results

We identified two overarching categories: “Making decisions about revision” and “Views about a randomised controlled trial” and a number of subthemes in each category. These two key categories and all subthemes within them are described in turn.

### Making decisions about revision

#### A balance of patient and disease characteristics

Surgeons based their decision to perform either a one-stage or two-stage revision on a balance between a complex mix of patient physiology (patient’s age, condition of bone and surrounding soft tissue), co-morbidities, cemented or un-cemented primary fixation, the patient’s preference and social situation, and the characteristics of the infecting organism (for instance whether they could identify the infecting organism and its antibiotic sensitivities):

For infection, if I know the organism, then I might consider – or they’re elderly where they can’t really survive without a hip, then there are situations where I would go with a one-stage. But otherwise, the default position is two-stage. (Dave)

My default is still two-stage, but if the patient’s young, and they’ve got a cementless hip […] and the patient comes in and has got a short-lived infection, I would think more towards one-stage. So, so, generally, I would say, if it’s a cemented hip, it gets a two-stage. If it’s cementless, it gets a one-stage. But then there are various things that will make me, veer the other way. For instance, a diabetic, I would generally do in two stages. (Tim)

Although surgeons made decisions based on patient characteristics and the results of investigative tests and radiographs before surgery, this decision could be revised intraoperatively:

If I’m doing a single-stage revision for infection, I would still warn the patient that if I find something untoward intraoperatively then my default position would be a two-stage. (Dave)

Sometimes in theatre, you get in there and you know, with an infected hip, quite often the bone quality is very poor […] And I think if the bone is soft, again, I would rather do a two-stage, if I can. (Tim)

If patients required a two-stage revision a temporary spacer loaded with antibiotics may be fitted for a period of typically 3–6 months. Alternatively the surgeon may choose not to use a spacer during this period. One surgeon explained that he thought that a two-stage strategy increased the likelihood of eradicating the infection by 10% but saw this as a marginal gain when compared to the prolonged treatment period and reduction in quality of life for the patient:

There’s a benefit of having two because you get that slightly higher, you know, but it’s only, you know, a 10 in 100 or whatever. It’s a, you know, or whatever it’s, you know, 80 versus 90% - most people understand that difference. […] They know it’s a marginal gain for a big investment because it’s not just a second operation. The main problem is for 2 or 3 months, they’re gonna be incapacitated. To say they’re stuck at home, unable to work, unable to get out and about, dependent on other people for everything […]

*That’s a long time isn’t it? Three months.*

It is if you’ve only got a few years left to live, yeah. (James)

Patients’ social and personal circumstances were also taken into consideration:

So if the patient is the sort of patient who’s going to do well with a pseudarthrosis of some sort, are they going to struggle using crutches, have they got home circumstances, because that might push you more towards considering a one-stage, if all the other factors are involved […] you may consider doing a one, even though it may not be quite what you want because of the patient factors, to improve their quality of life. (Saul)

#### Patients’ preferences

Surgeons reported that while some patients appear to be happy to follow their advice and rely on the surgeon’s decision, other patients had strong treatment preferences:

The other problem with doing a one-stage is trying to convince the patients because quite a lot of patients are coming along nowadays by the time they’ve come to see myself or any of the other consultants […] they have read up on infection and they know that there’s a higher incidence of recurrence in a single stage revision so very often they’re reluctant to have a single stage. (Duncan)

Patients could also elect not to have any further surgery if they felt they would prefer to go on long-term suppressive antibiotics:

After you’ve discussed the situation with the patient, discussed the potential for either a two-stage or a one-stage procedure, some patients elect not to go ahead. They’ve had enough of surgery. They may be elderly. They may be frail. They may simply want to go onto suppressive antibiotics, and accept the fact that the hip is not perfect. (Alex)

Surgeons also indicated that for older or frailer patients, the choice between surgery and long-term suppressive antibiotics was also determined by how the patient feels that their quality of life will be affected by the treatment.

#### The influence of infrastructure

The presence of physical and organisational structures and facilities needed to ensure operations can be performed are crucial. Surgeons suggested that deficits within the infrastructure could impact on treatment decisions. For instance, Duncan reported performing two-stage revisions because local microbiology services were unable to provide accurate service:

We didn’t until recently have the appropriate infrastructure in [our hospital] with regard to the microbiology department in order to be able to identify organisms accurately prior to surgery […] more often than not the aspirations would either come back negative or the laboratory would have some excuse as to why the sample hasn’t been tested properly […] Now over the last 2 years the microbiology department has changed and there’s a new set of individuals there who are more proactive and much more amenable to discussing matters with the clinician so it could well be that my view about [always doing two-stage] changes. (Duncan)

Costs to the NHS also played a role in treatment decisions. While the cost of performing a two-stage revision was considerably higher than a one-stage, it was seen to bring in more resources to the hospital:

Probably, the hospital gets paid more for doing a two-stage, right, which is always at the back of the surgeon’s mind […] the quality of patient care you can give, depends on your Trust not being in the red. So, you don’t want to do operations that lose your Trust money but your first priority is to get the best for the patient. Plus, when you’re in indecision, the fact that the two-stage operation doesn’t cost – that doesn’t, you know, it brings in more resources to the Trust, means you feel more relaxed about that option. (James)

At one hospital, the surgeons could not be sure that patients would have timely access to a bed for a second operation and so patients were kept in the hospital for the period between the first and second stage, increasing costs further:

When we start talking to hospital management they’re not very happy about two-stage procedures because it’s extra cost, extra time in hospital […] we usually do the second stage in about 8 to 10 weeks per surgery and we keep them in hospital for the whole period of time because we can’t guarantee that they will get readmitted at the specific time it was required. (Duncan)

Although it did not seem that economic costs influenced surgeons’ decisions, they nevertheless acknowledged some pressure to reduce costs. However a lack of access to adequate microbiology services presented a more direct concern and a barrier to the possibilities of performing one-stage operations.

#### Influence of literature, peers and training

Surgeons reported that they were influenced by published results of comparative studies of one- and two-stage revision surgery. They obtained this information at conferences and from scientific journals. Professional colleagues, particularly those more senior, also appeared to be influential:

[Consultant Q] himself is moving towards more one-stages and I think that’s sort of leaning more of us towards thinking about one-stages a, a bit more…and I think part of that is the results. […] We were all brought up, you know, 10 years, 15 years ago that two-stage is the gold standard and so on, but there’s more and more published results showing that one-stages are probably equivalent in terms of - in a lot of the cases in terms of microbiological clearance and so on, particularly if you’re careful that you pick the cases as we said, the more difficult ones. (Saul)

Otherwise everyone else has gone - pretty much gone for two-stage based on, I guess, the most published literature […] Although I, as you do as a registrar, I read all the one-stage papers – […] but they’ve certainly been pushed into the background compared to the two-stage papers, which have sort of dominated the literature for the longest period of time. So I end up doing two for most people. (Harrison)

#### Managing uncertainty

Of the 12 surgeons interviewed, six surgeons from three different centres used a custom-made articulating spacer (CUMARS). This consists of the same implants that would be used for a definitive reconstruction but that the surgeon chooses to cement (or fix) in more loosely than they would normally for a definitive primary or revision hip replacement [9]. Of the six surgeons who reported using this technique four suggested they used it almost exclusively (See Table 1).

Duncan reported that surgeons in the hospital where he worked had used a version of the CUMARS technique for around 15 years. However, although he used this method, most patients stayed in hospital for 8–10 weeks before receiving the second stage:

Yes we’ve been doing [the CUMARS] method for the last 15 years so it’s nothing new to us.

*Right, so basically all your two-stage are loosely cemented, and would you say if patients are happy with the first stage you would leave it with them?*

Well if they’re very elderly and infirm then yes we would. But that doesn’t happen often. (Duncan)

Tom explained that the CUMARS approach allowed him to modify how he fixed the spacer – which is an articulating ‘Exeter’ prosthesis – depending on his assessment of likely longer-term outcome; so a one-stage may remain as a one-stage in an older patient, whereas a younger patient may need a second revision later on:

So, if you were faced with an elderly patient who was very frail, you might try and do what is essentially a one-stage operation, using the spacer device to walk on and fix it as well as you could, and maybe that patient will never come to a second stage. A younger patient […] you might accept that it’s much more likely that you’re going to go to a second stage […] and so therefore, you would maybe not fix the hip quite so well and plan for a two-stage. So, it’s shades of grey rather than black and white situation and it’s a slightly odd approach too, the fixation of the implants. (Tom)

It appears that surgeons may use the CUMARS technique as it allows more flexibility in the longer term. This enables surgeons to leave open their options for further revisions, in the face of uncertainty about the definitive surgical solution and their uncertainty about certain patients’ outcomes.

Personal experiences of using CUMARS also prompted some surgeons to rethink their decisions about using one-stage or two-stage revision surgery:

It’s one’s personal experience with that, and seeing a reasonable number of people be happy with those spacers for a long time that, I guess will give you a bit of confidence to, just perform single-stage revisions. You know, if people are doing well after that procedure, you often wonder, ‘Well, if I had, just done things slightly differently, then they would have a better cemented implant, that is now likely to last an awful long time. And, why don’t I just stick with a single-stage, or tend to go more towards a second-stage?’ And that’s what I’ve tended to do, is drift in that direction. (Brian)

The surgeons who used CUMARS felt that it benefited patients, particularly outcomes such as pain and mobility. They also reported that use of an articulating implant avoided problems associated with cement spacers such as erosion of the acetabulum and pain:

The beauty of having a [CUMARS] is that essentially they’re happy, you know. They walk home. […] I’ve not had to feel sorry for a patient because they were struggling with pain because of something that I’ve put in. (Harrison)

Some of the cement spacers are, that are made, are designed to go in as articulating spacers up against the patient’s host acetabulum and those have got a terrible reputation for erosion on the acetabulum, pain, failure to allow patients to mobilise. (Tom)

### Views about a randomised controlled trial

#### A lack of evidence for superiority

When asked which technique they used most often, all surgeons reported that they most frequently performed two-stage revisions, and they defined CUMARS as a two-stage method. Surgeons quoted published re-infection rates for each of the two methods and thought that the two-stage method provided ‘slightly’ better outcomes, particularly in terms of eradication of infection. However, surgeons also questioned this, and reported some uncertainty in the light of the absence of comparative evidence:

My bias would be that, if I just intuitively if you said, ‘What are you doing for this patient?’ I’d just go, ‘two-stage’ because that’s what I’ve always done. That’s what I’ve been taught. That’s what I’ve read about. And that’s what appears to be slightly better. But I realise that scientifically there’s no big comparative trial. (Harrison)

Well, I suppose the truth is, we do not know which technique, one or two-stage, is better. We know the two-stage probably has a slightly better chance of eradication of infection than one-stage by about 10%, so that is the reason to do it. (Steven)

#### A shift in practice

While some surgeons felt that two-stage revision provides a slightly better chance of eradicating infection, others suggested that over time they had seen increasing evidence that one-stage revisions were equally effective and that this would be the ideal way to treat PJI. Reasons for this included improved surgical techniques, increasing evidence for the effectiveness of one-stage revision and the influence of colleagues’ successful results when using one-stage revision.

I did feel more comfortable with a two-stage revision generally and always have done, but increasingly over the last couple of years I have thought more about doing one stages […] and one colleague in the unit has done probably more one stages than the rest of us and seeing that his patients seem to be doing well. (Saul)

Ideally the best way would be to do the operations as a single stage with a very low recurrence rate. (Duncan)

I’m getting an impression that there’s increasing evidence that it’s [one-stage] an equally successful way of eradicating the infection in an awful lot of people. That it’s as good. (Brian)

I’m up for change. And this study is quite timely, because I think there is a general shift, at a lot of the meetings that we go to, with some people doing more one-stages, I think, than they used to […] I think we’re getting better at our surgery. […] I think with OSCAR [ultrasonic cement removal], you’re more confident about getting your cement out. (Tim)

Those who talked about their change in attitude most often referred to increasing published evidence and the observed success rates of their colleagues as their main influence. We did not observe that years of experience or caseload had influenced a shift in willingness to consider more one-stage revisions amongst surgeons who spoke about this, compared to those who did not. However, we did not ask surgeons directly if they felt their years of practice or caseload had influenced their decision-making with regard to one and two-stage revision for infection.

#### A future trial and equipoise in principle

All surgeons felt that a future randomised controlled trial comparing one-stage with two-stage revision is needed.

At the moment what we’re doing is not randomising and unfortunately rationalising to say that ‘this’ is better than ‘this’, but there are no good studies. So we need the evidence. (Dave)

To conduct an ethical randomised trial, there must be clinical equipoise in place [30]. Clinical equipoise is a state of uncertainty within the clinical community about the relative therapeutic merits of treatment options. Eligibility of a patient for a randomised trial depends on the existence of uncertainty about the best treatment option for that patient. When asked if they would be able to identify patients as eligible for randomisation in a trial comparing one-stage with two-stage revision surgery for infection, the majority were willing to put patients forward for randomisation when they were not convinced there was a best clear option, and where clinical equipoise existed.

*So you’re quite happy to randomise patients if they’re suitable?*

Yeah, but only obviously where I’m not convinced that there’s a clear best option. (James)

I’ve got a fairly open mind to that. […] Whilst in the majority of patients I guess I would be prepared to randomise them, there are, you know, occasional cases where you might feel that it’s not the right thing to do. (Brian)

*How would you feel about randomising your patients into one type of surgery or another?*

I’d, I’d be fairly happy about that, I think. I mean, if, er, unless there’s an obvious, you know, well, as long as there’s an opt-out in cases that you feel extremely strongly about, which there normally is [hmm] in these sorts of studies. (Howard)

I think it’s a good idea, but I think it’s actually very difficult, because, the patients are all so different [yeah], you know. It’s not just their, the infection. It’s the organism type they’ve got. It’s their age. It’s their comorbidities. It’s their ability to deal with a two-stage rather than a one-stage procedure. (Alex)

Some surgeons were asked what proportion of patients with PJI they would be happy to randomise to a trial comparing one-stage with two-stage revision surgery for infection. The majority of those asked, stated they would be happy to randomise most patients:

*Right okay, what proportion of patients that you treat for infection each year would, would you be happy to randomise do you think?*

All of them. (Duncan)

But in general, yes, I think I would be happy to randomise most of them. But yes, certainly, and the, the nasty gram-negatives, no, but otherwise most of them, yes. (Tim)

I think for, for a lot of the infections I would, I would be fairly happy to put most of them in, you know, a lot of them into that. Fortunately there aren’t huge numbers who fall into the really difficult bone or soft tissue category. (Saul)

## Discussion

**Fig. 1:**
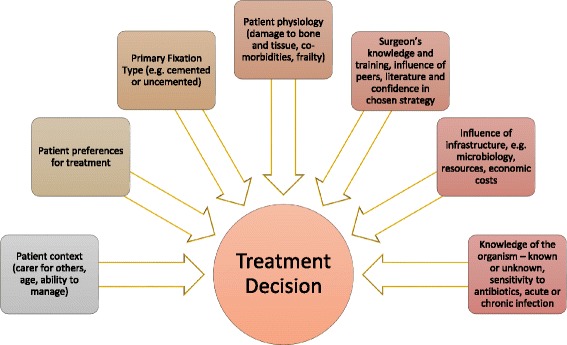
Factors involved when choosing a surgical strategy for the treatment of prosthetic joint infection

Our study highlights that surgeons balance multiple factors when choosing a surgical strategy for treatment of PJI. Factors included possible outcomes, multiple patient-related factors, their own knowledge and expertise, available infrastructure and the infecting organism (see Fig. 1).

Findings also indicate that there is no standard surgical response to PJI and surgeons try to find the ‘best fit’ for each individual. The absence of a strong evidence base makes any well intentioned attempt at developing more specific guidelines on using one-stage and two-stage revision surgery for PJI a difficult, if not impossible task.

Surgeons suggest that their willingness to consider a one-stage revision for patients with PJI has increased over recent years. With the more widespread adoption of a CUMARS approach, more efficient microbiology services, and more publicised research results that show equally favourable results for one-stage revisions, some surgeons are re-considering how they choose between one-stage or two-stage revision. This may relate to the trend seen in National Joint Registry data, which shows a modest but consistent year-on-year increase in the proportion of one-stage procedures conducted for PJI [31]. We suggest that the use of CUMARS enables surgeons to leave their treatment options open, while trying to reduce the negative impact of treatment on patients’ well-being. It seems possible that the use of CUMARS has prompted orthopaedic surgeons to rethink the debate about one-stage versus two-stage revision surgery. The study suggests that surgeons use CUMARS as a way of managing uncertainty in the absence of definitive evidence for the superiority of one surgical technique over the other.

There is a need for a randomised trial to compare one-stage with two-stage revision techniques to identify the most clinically and cost-effective treatment for PJI. Surgeons in this study were enthusiastic and welcomed the idea of a randomised trial. All surgeons described equipoise in principle for a selection of patients. While this might mean that recruiting enough patients to a trial of this kind may be challenging, it will be interesting to see whether the proportion of patients that surgeons consider to be eligible for randomisation changes over the course of the trial as surgeons become more comfortable with randomisation. Although one-stage revision was seen as the ideal way to treat PJI, surgeons thought that an absence of definitive studies meant that a change in practice was not yet warranted.

These findings compliment and build on our previous study of the impact of PJI and treatment on patients [10], which showed how PJI can adversely affect all aspects of patients’ lives causing physical and psychological distress. This echoes the concerns of surgeons’ in this study that decisions about the potential benefit of revision treatment – especially two-stage – must be balanced against the impact of that treatment on patients.

### Strengths and limitations

In this study, qualitative methods enabled us to characterise complex decision-making processes and the acceptability of a future randomised controlled trial. The sample size was small but achieved saturation and was appropriate to the qualitative study design [26, 28]. Specifically, the sample included surgeons with experience in one-stage and two-stage revision, from 5 different NHS hospitals. Robust thematic analysis ensured that saturation occurred, so that no new themes were identified in the later interviews. Although surgeons were asked to describe their decisions and we did not directly observe decisions being made in practice, or compare their decisions with hospital data, our focus was on how decisions were made and opinions about a future trial. As such, use of self-report was appropriate to the research question and approach.

## Conclusion

This article addresses an area in need of more research. Although clinical reviews are of great value [6, 32, 33], systematic reviews [16, 17] and the challenges faced by patients with PJI [10] highlight the need for strong evidence about one-stage and two-stage surgery. To design research that will impact on treatment we first need to understand reasons for current practice and assess feasibility of a future trial [22, 34]. Our study demonstrates that surgeons’ decisions are based on a complex combination of their own training and clinical experience, multiple patient-related factors, hospital infrastructure and the infecting organism. Our findings also show that some surgeons’ willingness to consider more one-stage revisions for infection has increased over recent years, influenced by better microbiology services, increasing evidence of equivalent effectiveness of eradication, and surgeons’ observations of colleagues’ success in using one-stage revision. We also find that the use of CUMARS has prompted orthopaedic surgeons to rethink their choice of revision surgery, and is possibly used as a way of managing uncertainty in the absence of definitive evidence for the superiority of one surgical technique over the other. Surgeons support the need to conduct a randomised trial comparing treatments for PJI. The next step is to conduct a robust, randomised trial to improve the evidence base for treating patients who develop a PJI after hip replacement.

## Additional file

Additional file 1: Infection after total joint replacement: Surgeon Topic Guide. Interview topic guide used by the researcher during interviews with surgeons. (DOCX 21 kb)

## Abbreviations

CUMARS: Custom-made articulating spacer
NHS: National health service
NRES: National Research Ethics Committee
PJI: Prosthetic joint infection
PROSTALAC: Prosthesis with antibiotic-loaded acrylic cement
RCT: Randomised controlled trial
