# Supplementary material for: Managing uncertainty - a qualitative study of surgeons’ decision-making for one-stage and two-stage revision surgery for prosthetic hip joint infection
Source: BMC Musculoskelet Disord. 2017 Apr 12;18:154. doi: 10.1186/s12891-017-1499-z (PMC5388991; doi:10.1186/s12891-017-1499-z)
Supplement: Additional file 1: — Infection after total joint replacement: Surgeon Topic Guide. Interview topic guide used by the researcher during interviews with surgeons. (DOCX 21 kb) [file 12891_2017_1499_MOESM1_ESM.docx]

Infection after total joint replacement: Surgeon Topic Guide

**Introduction and consent**

**Socio-demographic data**

Age/ years as a surgeon/ years treating infection after total joint replacement?

**Treating the infection**

- Which kind of hip revision operation do you perform most often (1 or 2 stage)?
- How many cases do you personally revise for infection each year?
- When do you make the decision to use either a one or two stage revision with a patient? (*At what point, before the operation, on the operating table?*)
- Take me through the decision making process that you go through when you decide to operate on a patient….*Who is involved? Is the patient involved?*
- What kinds of factors determine which kind of operation you would perform? (*Patient characteristics, context, staff available on the day?*)
- What kind of information do you give to patients about the treatment?
- Could you describe for me how you would explain the process to a patient in the language that you would use with the patient? *Do you feel they understand?*
- How do you feel about giving this information to patients? *Are there any challenges?*
- Do you tell patients about the two different kinds of revision surgery that are used to treat PJIs?
- Would you or have you ever asked a patient which treatment they would prefer? *Why/why not*?
- What kinds of questions do you have most often from patients about the treatment and care?
- How do you feel about treating patients at the [name of centre] (*is there anything about the area or the hospital itself that you think influences your experience or the patient experience of treatment*)
- What are your concerns for patients who have had infected hip replacements and who undergo revision surgery?
- What outcomes would define a successful treatment? For you and for the patient
- What impact does it have on you as both as a person and as a professional when a patient you have treated develops a deep prosthetic joint infection?

**Outcomes post revision surgery**

- What are your greatest concerns for patients during their recovery from revision surgery?

**Views on future research**

Because we don’t know yet which kind of treatment for prosthetic joint infection is best we hope in the future to do more research comparing surgical treatments for infection to see which is best and which one patients prefer.

- How do feel about randomising your patients into one type of surgery or another? (*Acceptability*?)
- What proportion of patients that you treat for infection each year are you happy to randomise?
- When would it not be acceptable to randomise a patient?
- How would you broach randomisation with a patient? How would you explain it to them? (Is that the language you would use?)
- If you were to randomise a patient how much of their treatment would you be happy to be defined by a protocol (e.g. antibiotic type and period, spacer, fixation)?
- As a surgeon and health professional taking part in this kind if research what sort of information do you want to know about the research?
- What are your feelings about the 1 and 2 stage revision treatments for joint infection? (*benefits/ disadvantages/ concerns*)
- What would you feel are the risks and benefits to you of taking part in research?
- Do you have any preconceptions of what you think the results of the study might be?
- If a clear benefit is demonstrated for one treatment over the other, would it change your practice?

**About participating in this study**

Is there anything about your participation in this research that you would have liked to have happened differently?

**Conclusion**

- Is there anything else you would like to add, or anything you wish to talk about that we haven’t covered already?
- Would you like us to send you a brief report of the study findings?

**Reaffirm consent…thank you for participating…END.**
